# Evaluating the effect on asthma quality of life of added reflexology or homeopathy to conventional asthma management – an investigator-blinded, randomised, controlled parallel group study

**DOI:** 10.1080/20018525.2020.1793526

**Published:** 2020-07-14

**Authors:** Ayfer Topcu, Anders Løkke, Leila Eriksen, Lars Peter Nielsen, Ronald Dahl

**Affiliations:** a Department of Medicine, The Regional Hospital in Horsens, Horsens, Denmark; b Department of Medicine, Little Belt Hospital, Vejle, Denmark; c Department of Regional Health Research, University of Southern Denmark, Odense, Denmark; d CAM Consultant, Reflexologist, Copenhagen, Denmark; e Department of Clinical Pharmacology, Aarhus University Hospital, Aarhus, Denmark; f Global Medical Expert, GSK, Copenhagen, Denmark

**Keywords:** Complementary and alternative medicine, reflexology, homeopathy, asthma, randomised controlled trial, quality of life

## Abstract

**Background:**

Asthma is a common chronic disease worldwide without any known cure. Despite remarkable improvement in asthma treatment, better education and guideline implementation strategies, there is growing interest in using complementary and alternative medicine, like reflexology and homeopathy. However, evidence supporting the effectiveness of homeopathy and reflexology in asthma treatment is not available.

**Objective:**

The aim of this study was to evaluate the effect of reflexology and homeopathy as adjunctive therapies in asthma.

**Methods:**

In a single centre, randomised, investigator blinded, controlled study 86 asthma patients were enrolled. They were assigned to one of three study groups (conventional treatment alone or conventional treatment with addition of either homeopathy or reflexology). All patients received their asthma treatment during the study and were followed as usual by their general practitioner. The study assignment group of individual patients were blinded to the investigators, who made the clinical evaluation of asthma control. The primary outcome was the change in the asthma quality of life questionnaire (AQLQ) scores after 26 weeks. Secondary outcomes included asthma control questionnaire, EuroQol, forced expiratory volume in 1 sec, morning and evening peak expiratory flow, asthma symptoms, rescue medication use, and total medication score.

**Results:**

Minor improvements in the AQLQ score were observed in all three groups. However, no statistically significant changes in AQLQ scores were seen within or between groups. Likewise, secondary outcomes did not differ between groups.

**Conclusions:**

In this study, the addition of homeopathy or reflexology to conventional treatment did not result in improved quality of life in asthma.

## Introduction

Patients with chronic diseases such as asthma are among the most frequent users of complementary and alternative medicine (CAM) [1,2]. Studies show that the prevalence of CAM use in asthma and rhinosinusitis varies from 27.2 to 59% [3–7].

A recent survey of American Academy of Allergy, Asthma and Immunology members investigating patterns of CAM use and adverse effect from CAM showed 81% of respondents had patients who are using CAM therapies and 80% of respondents were interested in learning more about CAM [8]. Another study in Canada showed that asthma and allergy were the most common diseases advertised by the majority of alternative healthcare clinics [9].

Homeopathy and reflexology are forms of CAM therapies frequently used by asthma patients [3]. Homeopathy is based on two main principles. These principles state that a substance, causing a certain symptom after intake, can be used in diluted form to treat a similar symptom in illness and that a more diluted substance is more effective [10,11]. It has been postulated that homeopathic substances stimulate auto-regulatory and self-healing processes [10]. There are different types of homeopathic interventions including classical, clinical, complex, and isopathic homeopathy [12].

Reflexology is as a method of treatment based on the principle that reflex zones corresponding to different organs are located underneath the feet and massaging particular areas of the foot can produce a therapeutic effect in corresponding organs and tissues [13].

Several trials have evaluated the effect of homeopathy and reflexology for asthma. However, the existing evidence for homeopathy and reflexology is insufficient to support their use as a supplement in asthma. Systematic reviews have found that clinical trials testing homeopathy and reflexology have major flaws, such as small number of participants, lack of control groups, or inadequate allocation concealment [14,15].

Therefore, the aim of the present study was to assess the effect of reflexology and individualised homeopathy as an adjuvant treatment in asthma. In order to address this issue, we conducted an investigator-blinded, randomised, controlled parallel group study.

## Methods

### Patients

Patients were recruited from our outpatient clinic, from general practitioners and by advertisement in local newspapers. Eligible patients were at least 18 years of age and had a history of bronchial asthma for minimum 6 months prior to baseline. For inclusion, patients had to have a forced expiratory volume in 1 sec (FEV_1_) ≥ 60% predicted before bronchodilator and an objective measure of abnormal variation in bronchial calibre [16]. This variation could be at least one of the following characteristics: (1) a positive bronchodilator reversibility test defined as increase in FEV_1_ ≥ 10% after 400 µg inhaled salbutamol; (2) a positive methacholine challenge test defined as a PD_20_ of < 1000 μg; (3) a positive test for exercise-induced asthma defined as a fall in FEV_1_ > 15% after a standardised 6-min exercise test; or (4) a positive peak expiratory flow (PEF) variability defined by ≥ 3 days or 2 consecutive days with a differences between morning and evening PEF of > 20% during a 2-week period.

Patients were excluded if they had been hospitalised for asthma within 3 months prior to inclusion, or if they had an asthma exacerbation during the last month. Other exclusion criteria included changes in asthma medication within 30 days of screening and a smoking history > 10 pack years.

### Study design

This was a randomised, investigator blinded, controlled, parallel-group study. The protocol was approved by the Aarhus County Committee on Biomedical Research Ethics and the Danish Data Protection Agency. All subjects provided written informed consent before participating in any study-related procedures. The study was conducted according to Good Clinical Practice standards and was monitored by the Good Clinical Practice Unit at Aarhus University Hospital. A steering committee consisting of respiratory specialists and registered practising homeopath and reflexologist met regularly and were responsible for conducting the study and the cooperation between the involved professionals.

**Figure 1.**
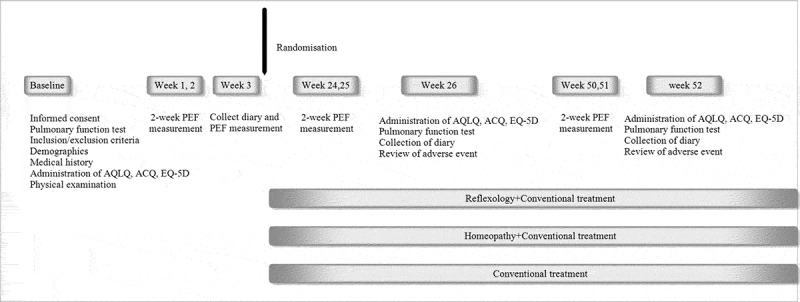
Study flow chart.

After a 2-week screening period, eligible patients underwent randomisation. Figure 1 demonstrates the flowchart of the six planned visits during the study period.

The screening visit included a medical history of asthma, spirometry, blood sample testing for eosinophils, eosinophil cationic protein (ECP), and specific IgE to common inhalant allergens, assessment of quality of life, evaluation of asthma control, instruction in the use of peak flow meter, and filling in a diary card. During the following 2-week period patients filled in a symptom diary every morning and evening including peak flow measurement and rescue medication use. After the 2-week run-in period, additional tests were performed on an individual basis to reveal significant changes in FEV1 after exposures to confirm the presence of asthma. Eligible patients were randomised to one of three groups: conventional treatment alone, conventional treatment plus reflexology, or conventional treatment plus homeopathy. Baseline data were collected from screening visit, during the run-in period of 2 weeks and randomisation visit. Symptoms and PEF were monitored again for 2-week periods before clinic visits at weeks 26 and 52. All other baseline assessments were repeated after 26 and 52 weeks. Visit at week 26 was divided in two parts. The first part consisted of spirometry and reversibility, blood sample testing for eosinophils, ECP, assessment of quality of life, evaluation of asthma control, and collection of diary card. The second part occurred 1–4 days after first part to assess bronchial hyper-responsiveness (BHR) by performing a methacholine challenge test (data not shown here). At week 52, the procedures were the same as at week 26.

The patient’s general practitioner was informed about the study participation and during the study, patients were instructed to contact their usual healthcare providers regarding asthma management including prescription of asthma medications and treatment of acute asthma exacerbation. To mimic a real-life situation, caregivers did not receive any study-related asthma education sessions or materials.

### Interventions

Patients in the conventional treatment group received usual care of asthma. This treatment was monitored and adjusted as usual by the patient’s general practitioner. Patients in the homeopathy group received homeopathic treatment in addition to usual care of asthma. Homeopathic treatment was performed by a registered homeopath and based on principles for classical homeopathy. The therapist was appointed by the Danish Society of Classical Homeopathy. Homeopathic treatment was decided on an individual basis by the homeopath and prescribed as an oral treatment. Patients received homeopathic product with potency between C30 (dilution by a factor 100^30^ = 10^60^) and M10 (dilution by a factor 1000^10^ = 10^30^). The number of homeopathy sessions attended was 6–12 during 1 year.

Patients in the reflexology group received reflexology treatment in addition to usual care of asthma. Reflexology was performed by one of two registered reflexologists. The therapists were appointed by the Danish Reflexology Association. Patients generally received treatments weekly for 4–6 weeks, followed by two treatments during 1 month. Treatments were then given monthly until the end of the study. The sole of the foot was treated by applying pressure along each reflex zone. After that, gentle pressures were applied to one or more zones and to zones which reflected energy imbalance or blockage. Patients were also given advice about self-care according to opinion of the reflexologist.

### Effect assessments

#### Assessment of quality of life

Asthma quality of life questionnaire (AQLQ) is a self-administered asthma-specific questionnaire which consists of 32 questions in four domains (symptoms, activity limitation, emotional function, and environmental stimuli) [17]. The overall AQLQ score is the mean of all 32 questions. A change in score of ≥ 0.5 is accepted as the minimal clinical important difference (MCID) in AQLQ [18].

Asthma control questionnaire (ACQ) includes seven questions, five of which concern symptoms and activity limitation, one about short-acting β_2_-agonist use and one about FEV_1_% predicted [19]. The question, concerning FEV_1_% predicted, was completed by clinic staff. Patient recalled their experiences during the previous 7 days and responded to each question using a seven-point scale (0 = totally controlled to 6 = extremely poorly controlled). The items are equally weighted and the ACQ score was the mean of the seven items. A change in score of ≥ 0.5 is accepted as the minimal clinical important difference in ACQ [20].

EuroQol-5D (EQ-5D) is a self-report questionnaire, which measures general health [21]. The five questions have three levels of severity (no problem/some problem/extreme problem). The resulting health status can be defined by a five-digit number by combining one level from each of the five dimensions. This measure is referred to as EQ-5D_index_. In addition, the patients are asked to rate their present health status on a vertical graduated (0–100) visual analogue scale. This is referred to as EQ-5D_VAS_ [21]. A change of 0.074 or more is accepted as a relevant clinical difference in EQ-5D_index_ score.

#### Asthma symptoms, pulmonary function tests

Morning and evening PEF, asthma symptoms, and rescue medication use were recorded in patient diaries 2 weeks prior to weeks 2, 26, and 52. Asthma symptoms were recorded on scales from 0 to 5 for daytime symptoms (0 indicating no symptoms and 5 disabling symptoms) and from 0 to 4 for night-time symptoms (0 indicating no symptoms and 4 symptoms causing wakefulness all night). Morning and evening PEF (Mini-Wright peak flow meter, Clement Clark Ltd, Harlow, UK) were performed before inhaled medication and the highest of three reproducible measurements was recorded.

Spirometry was performed according to the American Thoracic Society recommendations [22] and predicted values of the European Coal and Steel Community [23] were used. FEV_1_ and forced vital capacity were recorded using a dry wedge spirometer (Vitalograph®, Buckingham, UK) at baseline, week 26 and week 52.

#### Atopic status

Patients were tested for specific sensitisation to inhalant allergen by serum measurement of specific IgE with the ImmunoCAP system (Phadia AB, Uppsala, Sweden). The six inhaled allergens tested were dog, cat dander, grass, mugwort, birch pollen, and house dust mite.

#### Total medication score

Total medication score was the sum of the scores given to each prescribed controller and reliever medication. Medication score for inhaled corticosteroids was calculated by converting inhaled corticosteroid doses to beclomethasone dipropionate equivalent doses (1 point: inhaled corticosteroid (ICS) ≤ 500 µg, 2 points: 501 µg ≤ ICS ≤ 1000 µg, 3 points: 1001 µg ≤ ICS < 2000 µg, 4 points > 2000 µg, 5 points: oral steroid). Calculations were based on GINA estimation of equipotency of inhaled corticosteroid in metered doses: fluticasone propionate 500 µg = budesonide 800 µg = beclomethasone diproprionate 1000 µg [12]. One point for each of the following medications was given: short-acting β2-agonist, long-acting β2-agonist, leukotriene modifier, theophylline, inhaled short- and long-acting anticholinergic. All individual scores were summed for a total score which could range from 0 to 10.

#### Exacerbations

An exacerbation was defined as worsening of asthma symptoms that led to use of systemic corticosteroids and/or antibiotics. The use of antibiotics and corticosteroids was recorded by patients in a diary.

#### Adverse events

All adverse events were recorded during the study.

### Trial endpoints

Quality of life measured by the AQLQ was the primary endpoint. AQLQ was evaluated as the change from baseline after week 26 and week 52.

Secondary endpoints included quality of life measured by the ACQ and EuroQol (EQ-5D), spirometry, use of medication measured as a standardised medication score, asthma symptoms, and PEF monitoring during a 2-week period and rate of exacerbation. The change from baseline was evaluated after 26 and 52 weeks.

### Statistics

#### Randomisation and masking

Randomisation was based on a computer-generated block randomisation list and performed independent of the research team. A member of the clinic staff, who was otherwise not involved in the study, gave the treatment allocation code to the patient and secured the referral to the related therapist. The allocated treatment group was unknown to the investigators. Subjects were discouraged to reveal their allocation to the investigational staff.

#### Sample size and statistical analysis

Prior to the study, it was calculated that 40 patients per treatment group were required to detect a 0.5 point change in AQLQ score with an 80% power and a two-sided 0.05 α-level test.

The analysis population for all efficacy end points was the intention-to-treat (ITT) population, which included all randomised subjects who had received at least one treatment session. Additionally, the last observation obtained from a patient was substituted for all subsequent observations that were missing (Last Observation Carried Forward).

Intercooled STATA (version 11, Stata Corporation, Collage Station, Tx, USA) software was used for statistical analysis. Mean and confidence interval (CI) was calculated for variables with normal distribution. Analysis of variance was used to estimate treatment group means and between-group differences. In case of non-normal distributions, the median with minimum and maximum ranges was calculated. These variables were analysed by Kruskal-Wallis test.

## Results

### Study population

Of 187 patients screened, 89 patients did not fulfil the inclusion criteria. The most frequent reason for ineligibility was absence of an objective measure of abnormal variation in bronchial calibre.

**Figure 2.**
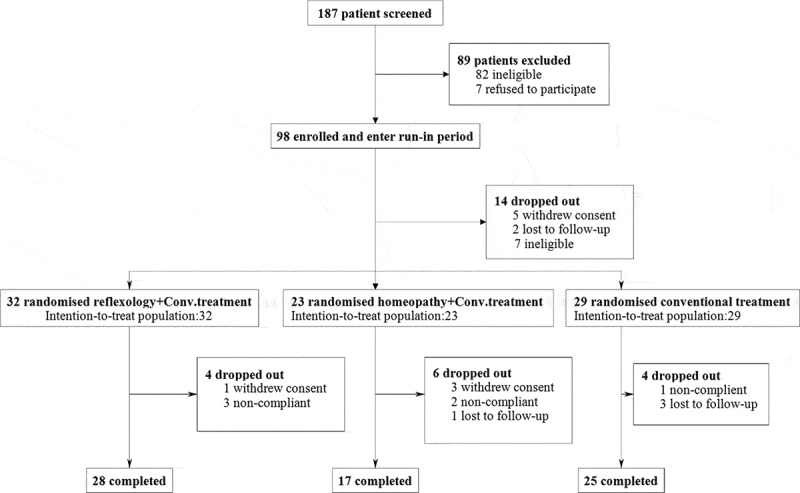
CONSORT diagram of the trial profile.

About 98 patients were enrolled and 84 were randomised to one of the three treatment groups. A total of 14 patients dropped out during the study (reflexology *n* = 4, homeopathy *n* = 6, and conventional treatment *n* = 4). Data from 84 patients were used for statistical analysis. Figure 2 shows a CONSORT diagram of the trial flow.

Demographic and asthma characteristics of the subjects at baseline are listed in Table 1. No significant differences were observed between groups at baseline.

**Table 1.** Baseline demographic and asthma characteristics of the 84 subjects randomised in the study (ITT population).

	Reflexology + conv. treatment *N* = 32	Homeopathy + conv. treatment *N* = 23	Conv. treatment *N* = 29	*p* value
Gender Female/Male, *n*	19/13	16/7	19/10	0.81
Age [years] Mean, range	47.7 (20–79)	40.3 (18–67)	44.6 (19–79)	0.25
Atopy, *n* [%] (positive result in Phadiotop phanel)	19.0 (59.4)	17 (73.9)	23 (79.3)	0.38
Morning PEF(L/min) Baseline (during the run-in period of 2 weeks), mean ± SD	424.2 ± 126.9	431.4 ± 102.1	428.1 ± 82.9	0.97
Evening PEF (L/min) Baseline (during the run-in period of 2 weeks), mean ± SD	431.4 ± 118.3	436.1 ± 100.6	439.6 ± 88.4	0.95
FEV_1_ (L) Baseline, mean ± SD	3.0 ± 0.9	3.1 ± 0.8	3.0 ± 0.8	0.90
FVC (L) Baseline, mean ± SD	3.95 ± 1.2	4.0 ± 1.1	3.95 ± 0.9	0.98
AQLQ Baseline, mean ± SD	5.9 ± 0.9	6.0 ± 0.9	6.0 ± 0.9	0.95
ACQ Baseline, mean ± SD	1.2 ± 0.8	0.9 ± 0.8	0.9 ± 0.7	0.48
EQ-5D_VAS_ Baseline, mean ± SD	81 ± 15	75 ± 16	84 ± 11	0.06
EQ-5D_index_ Baseline, mean ± SD	0.89 ± 0.14	0.91 ± 0.12	0.92 ± 0.11	0.71
Total medication score Baseline, mean ± SD	3.47 ± 1.3	3.48 ± 1.3	3.55 ± 1.2	0.96

### Primary endpoint

**Figure 3.**
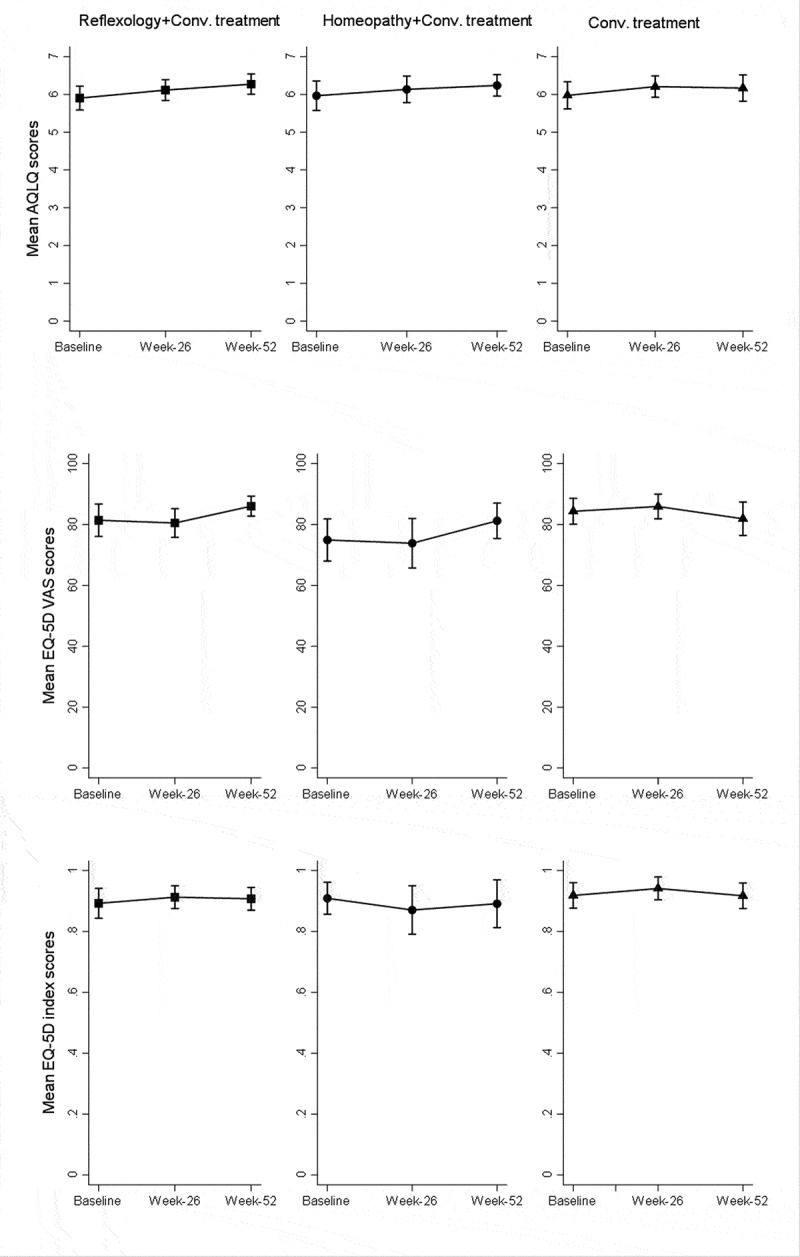
Mean AQLQ _scores_, EQ-5D_VAS_ and EQ-5D_Index_ scores at all time points (Error bars represent 95%CI).

The AQLQ score was improved in all study groups from baseline to week 26. Subsequently, the AQLQ score in reflexology and homeopathy groups continued to improve, meanwhile it stabilised in conventional treatment group at week 52. However, there were no statistically significant differences between groups in AQLQ neither at week 26 or week 52. Mean improvements from baseline to end of the treatment in AQLQ were lower than MCID in all groups and no statistically significant differences were observed between groups (Figure 3). The non-significant improvements in overall AQLQ score were paralleled in individual domain scores for activity, symptoms, emotional function, and environmental stimuli in all groups. However, there were no significant differences between groups (Table 2).

**Table 2.** Mean specific domain quality of life score for all groups.

		Reflexology + conv. treatment *N* = 32	Homeopathy + conv. treatment *N* = 23	Conv. treatment *N* = 28
	Baseline	5.7 [5.4; 6.1]	6.0 [5.5; 6.4]	6.0 [5.6; 6.3]
Symptoms	Week 26	6.0 [5.7; 6.3]	6.1 [5.8; 6.4]	6.2 [5.9; 6.5]
	Week 52	6.2 [5.9; 6.5]	6.2 [5.9; 6.6]	6.1 [5.8; 6.4]
	Baseline	5.7 [5.3; 6.1]	5.7 [5.2; 6.2]	5.5 [5.1; 6.0]
Environment	Week 26	6.0 [5.6; 6.4]	5.9 [5.4; 6.3]	5.9 [5.5; 6.3]
	Week 52	6.1 [5.8; 6.5]	6.0 [5.6; 6.5]	6.1 [5.7; 6.4]
	Baseline	6.2 [5.9; 6.6]	6.1 [5.6; 6.5]	6.2 [5.9; 6.6]
Emotions	Week 26	6.4 [6.1; 6.9]	6.2 [5.8; 6.5]	6.5 [6.2; 6.8]
	Week 52	6.5 [6.2; 6.8]	6.4 [6.1; 6.7]	6.5 [6.2; 6.8]
	Baseline	6.0 [5.7; 6.3]	6.0 [5.7; 6.4]	6.1 [5.7; 6.4]
Activity limitation	Week 26	6.2 [5.9; 6.5]	6.3 [5.9; 6.6]	6.2 [5.9; 6.5]
	Week 52	6.3 [6.1; 6.6]	6.3 [5.9; 6.6]	6.2 [5.9; 6.6]

*Means [95%CI]

Result for the intent-to-treat (ITT) analysis was consistent with that of the per-protocol (PP) analysis.

### Secondary endpoint

#### Asthma control questionnaire

Reduction in ACQ at week 26 was 0.09 (95% CI: 0.23; 0.51) among patients receiving reflexology, 0.13 (95% CI: 0.30; 0.03) those receiving homeopathy, and 0.19 (95% CI: 0.24; 0.06) in conventional treatment group. Reduction in ACQ score continued both in the reflexology and homeopathy group but not in the conventional treatment group at week 52. However, differences between or within groups did not achieve a clinically or statistically significant level neither at week 26 nor week 52.

#### EuroQol

General health, as estimated by the EQ-5D VAS score, decreased in reflexology and homeopathy at week 26. End of the study, VAS scores increased numerically in the homeopathy and reflexology groups but not in the conventional treatment group. However, no clinical or statistically significant differences in scores were observed between groups at any time point. EQ-5D index scores also did not differ between groups and none of the groups had a minimal important improvement in EQ-5D_index_ scores (Figure 3).

#### Symptom score, bronchodilator use, total medication score

No significant differences were found regarding total medication score, daytime or night-time asthma symptom scores at any time points neither compared to baseline nor between groups (Table 3).

**Table 3.** Median for asthma daytime symptoms, night-time symptoms, rescue medication, and total medication scores for all groups.

		Reflexology + conv. treatment *N* = 32	Homeopathy + conv. treatment *N* = 23	Conv. treatment *N* = 28
	Baseline	0.78 [0; 4.2]	0.43 [0; 3.86]	0.36 [0; 3.50]
Daytime asthma symptoms	Week 26	0.43 [0; 4.2]	0.23 [0; 3.57]	0.21 [0; 2.43]
	Week 52	0.14 [0; 4.2]	0.14 [0; 3.57]	0.29 [0; 2.50]
	Baseline	0.04 [0; 2.10]	0 [0; 1.14]	0 [0; 2.21]
Nocturnal asthma symptoms	Week 26	0.07 [0; 2.10]	0 [0; 0.57]	0 [0; 1.57]
	Week 52	0 [0; 2.10]	0 [0; 0.57]	0 [0; 1.50]
	Baseline	0.11 [0; 5.77]	0.07 [0; 3.23]	0.29 [0; 3.00]
Rescue medication use (puff/day)	Week 26	0.1 [0; 5.36]	0.21 [0; 1.07]	0.21 [0; 4.00]
	Week 52	0 [0; 5.21]**	0.07 [0; 2.00]	0.21 [0; 3.00]
	Baseline	4 [1; 7]	3 [1; 6]	3 [1; 6]
Total medication score	Week 26	3 [1; 7]	4 [0; 6]	4 [1; 6]
	Week 52	3 [0; 7]	3 [0; 5]	3 [1; 6]

*Median (min, max); **p < 0.05.

Use of rescue medication from baseline to week 52 significantly decreased in the reflexology group from a median of 0.11 to 0 puffs/day (*p* = 0.03). The reduction in the control group from a median of 0.29 to 0.21 was not significant. In the homeopathy group, no difference was observed from baseline to week 52 in rescue medication use. The difference between treatment groups was not statistically significant (Table 3).

#### Peak expiratory flow and spirometry

**Figure 4.**
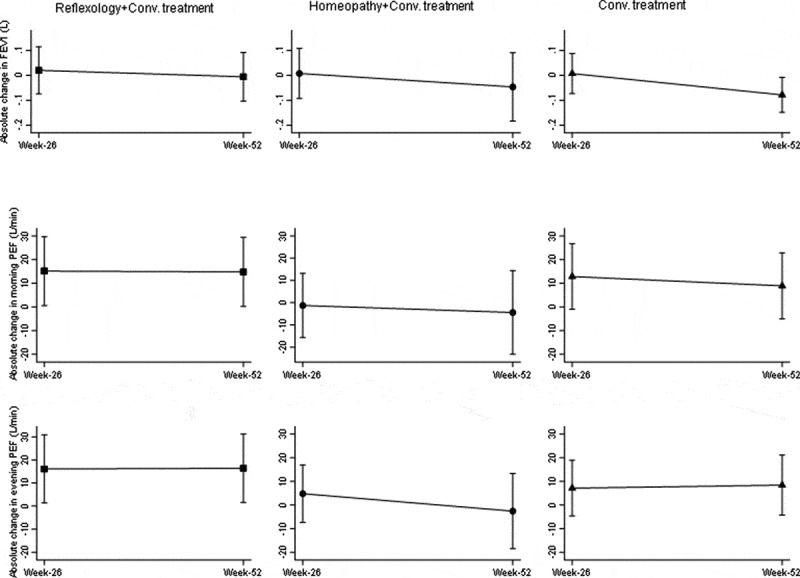
Absolute change in PEF and FEV_1_ at week 26 and 52 (Error bars represent 95%CI).

The absolute change from baseline was not significantly different between groups with respect to morning, evening PEF and FEV_1_ at week 26 and week 52 (Figure 4).

#### Exacerbation

The median rate of exacerbations at week 26 among patients assigned to reflexology was 0.17, as compared with 0.1 among those assigned to homeopathy and 0.12 among those assigned to control group. The rate of exacerbation was not different between groups. Exacerbation ratio reduced end of the study in both the reflexology and control group but not in homeopathy group. However, the difference between groups was not significant.

#### Adverse events

Most adverse events were mild or moderate. Five serious adverse events (SAEs) were recorded during the study. Three were reported in the reflexology group, two of which were asthma exacerbations. One was reported in both the homeopathy and the control group. No difference was observed between groups. None of the SAEs were suspected to be intervention related. There were no discontinuations caused by asthma exacerbation.

#### Adherence to study

About 28 patients (88%) in reflexology, 17 in homeopathy (68%), and 25 in conventional treatment group (86%) completed the study. Withdrawal differences between groups were not statistically significant.

## Discussion

This prospective, randomised, controlled study assessed the effect on asthma quality of life of reflexology and homeopathy when added to conventional treatment in asthma. The study showed no influence of homeopathy and reflexology on asthma outcomes. The outcome evaluations included asthma-related and generic quality of life, tests for asthma control, asthma symptoms, rescue medication, peak flow measurements during 2-week periods of diary home recordings, clinic spirometry, and the rate of exacerbations.

The study was performed in a single centre and followed guidelines for good clinical practice. Patients were randomly assignment to interventions and they were well characterised according to guidelines for diagnosis and classification of asthma. A total of 74 patients (88%) had a positive reversibility test, defined as an increase in FEV_1_ ≥ 12% and ≥ 200 ml after inhalation of 400 µg salbutamol. Only four patients had positive reversibility test, defined as increase in FEV_1_ ≥ 10% after inhalation of 400 µg salbutamol but these patients had moderate to severe BHR at baseline, even though they were on anti-asthmatic treatment. The baseline characteristic, e.g. age, sex, spirometry, and asthma severity were comparable between groups.

The present study was designed to reflect treatment practise, as patients were allowed to use all kinds of medications prescribed by their general practitioner and CAM treatments were used as add-on to patients that were symptomatic on existing treatments. Moreover, we deliberately designed our study without attempting to introduce placebo treatments as especially reflexology placebo treatment has proven very difficult and in fact introducing study bias [24]. Another important part of our study design was length of the follow-up period. Most of earlier studies investigating effect of homeopathy and reflexology in asthma lacked a long-term follow up. A shorter intervention might give a larger risk of a placebo response, which is often present in the first few months of an intervention. The 1-year follow up was a duration that ought to allow for a sustained response to be seen in our study.

The reason for selecting AQLQ as a primary outcome was to provide validated evidence of health from the patient perspective. Due to the obvious value of patient’s experience of treatment, use of patient reported outcomes is advised for measuring treatment effect [25]. This primary outcome parameter was acceptable for all involved in the study. The CAM therapies used in the present study are based on a holistic approach and have adopted a ‘whole system of care’ approach. In addition to the asthma-related AQLQ as primary endpoint, the ACQ and the generic quality of life questionnaires EuroQual 5D were used. This would enable detection of influences of the CAM interventions health-related quality of life as well as on general health status. However, no differences between groups were seen in any of the health-related or generic quality of life questionnaires. Minor improvements in the AQLQ score were seen in all three groups. There was no statistically significant change within or between groups although patients in all three groups had room for further improvement.

The result of our study is consistent with published randomised controlled studies with acceptable quality and intervention. Brygge et al. compared the efficacy of reflexology and placebo reflexology in 40 asthma patients before and after 10-week intervention [24]. Both groups showed improvement in quality of life, symptoms, and bronchodilator usage but differences between the two groups were not statistically significant.

Lewith et al. compared homeopathic therapy with placebo in 242 adults with asthma and allergy to house dust mite during a 20-week period [26]. After a 4-week run-in period, patients received an ultra-molecular dilution of house dust mite extract or placebo orally as three doses given in 24 h. Significant improvement in quality of life and FEV_1_ was reported in both group but differences between the treatments failed to achieve significance. No differences were found between groups in other outcomes at the end of the study. However, the study only considered intervention against house dust mite allergy.

Individualised homeopathy in asthma was studied by White et al. [27]. About 96 children with mild to moderate asthma were in a double-blinded fashion randomised to receive individualised homeopathic treatment or placebo for 1 year. No differences were observed between active and placebo-treated patients in quality of life (primary end point), peak flow, medications, and days-off school. The result is in accordance with our finding in adults and the study had sufficiently long observation time to evaluate clinical outcomes.

More recently, Thompson et al. compared usual care and usual care plus individualised homeopathy in 39 children requiring second care for asthma by using a mixed-methods protocol with quantitative and qualitative component. Children in the homeopathy group had five visits package of homeopathic care during the 16 weeks. The quantitative clinical outcome measures (ACQ, the paediatric asthma quality of life and medication change questionnaire) showed no benefit of homeopathic therapy over usual care. However, according to qualitative data most families believed that homeopathic treatment had improved the child’s asthma symptoms. On the other hand, this study did not collect quantitative data from usual care arm or those patients who withdrawn from the study after randomisation [28].

On the basis of reported CAM use in asthma, we had expected a larger interest from patients to participate. However, recruitment into the trial was lower than anticipated from the sources that were tried to interest patients to take part, i.e. from the patients attending the outpatient clinic, from announcements in general practice, and local advertising in newspapers and television. For the patients participating in run-in evaluation, the most common reason for failure to qualify for inclusion was the lack of the objective criteria for asthma. Patients may have abstained from attending because they could not select the treatment group themselves but were allocated by randomisation. Unfortunately, pre-mature closure of inclusion to homeopathy arm resulted in lower patient number in this group. Despite extensive efforts, a lower than expected number of patients was enrolled in this study and the negative results in our study could be interpreted in the context of whether these might be due to type-II errors. However, in our study the randomisation ratio was lower. Unequal randomisation ratios will only significantly reduce the power of a study if the ratio is 3:1 or more [29]. Additionally, the adherence to study procedures was high during this 1-year study. However, no study parameter provided any indication that significant differences would have been present even in a larger study population.

The responses on AQLQ at baseline in our study showed that asthma caused only little limitation to their quality of life. This can of course make it very difficult for any intervention to result in any further increase in quality of life. Moreover, a total of 62 patients (74%) had adequate asthma control (ACQ score < 1.5). Present study used neither AQLQ nor ACQ score to define as eligibility criteria. This was the limitation of our study. However, as an expected result, participants of the present study were included mostly from primary care population. Furthermore, recruiting patients with severe asthma would have done recruitment into the study even more difficult. Besides, Thompson et al. investigated effect of homeopathy in 38 children suffering from severe asthma in secondary care and their result did not show any evidence in favour of adjunctive homeopathic treatment [28].

Other types of CAM therapies, like acupuncture, chiropractic care, and breathing exercise have been examined in asthma. Breathing exercise as a non-pharmacological approach is mentioned in the current asthma guidelines and it is concluded that breathing exercise may be a useful supplement to asthma pharmacotherapy [16]. A Cochrane review reported breathing exercises for asthma such as Buteyko, yoga, and diaphragmatic breathing can reduce asthma symptoms and led a trend for improvement in quality of life [30].

The holistic nature of CAM and focus on patients’ individual needs and resources may be the explanation for the increasing interest and awareness of CAM, among patients with asthma. The other possible motivating factors for choosing CAM can be poor asthma control and quality of life or patients want to try all available healthcare options. As present asthma therapies are mainly symptomatic and not curative, many patients look for possibilities for cure of diseases that the established medical community cannot provide. Furthermore, CAM therapies have gained a support from the society including positive statements from people with influence on public opinion. Therefore, evaluation of CAM therapies is of interest for chronic disease management. However, the attitude to disease management must be based on evidence rather than opinion. It is important to have CAM therapies evaluated according to the same principles as other disease interventions. Randomised clinical trials should be the key action to establish efficacy.

Moreover, mechanism of action for both homeopathy and reflexology is very poorly understood. We have no information about such therapies affect the underlying pathophysiology of the asthma. Therefore, future CAM research should address questions of possible effect mechanism in disease states. Positive treatment outcome from such studies lead to the acceptance of homeopathy and reflexology in asthma treatment.

Furthermore, a small number of studies have addressed economic issues related to the cost effectiveness of CAM in asthma. A study evaluated cost effectiveness of homeopathy plus usual care in comparison with usual care in children requiring secondary care for asthma and national healthcare costs were significantly higher in the homeopathy group [28]. However, recent review of the economic evaluations of homeopathy concluded that variability and limitations in methodology had a significant impact on their ability to interpret these studies, although the identified evidence of the cost seemed promising [31]. A lower cost of care can also make these therapies attractive but first of all beneficial effects have to be established before such calculations can be performed.

In summary, the present study has not provided evidence for clinical effects by addition of homeopathy or reflexology to conventional treatment in asthma. This outcome of the influence of reflexology and homeopathy supports the results of the few others publish properly controlled studies in asthma.
